# Tuberculosis and Neoplasm of the Bladder; Surgery or Hygiene?

**Published:** 1896-04

**Authors:** 


### TUBERCULOSIS AND NEOPLASM OF THE BLADDER. SURGERY OR HYGIENE?

At the recent meeting of the American Association of Genito-Urinary Surgeons (*Med. News*, June 22d), L. Bolton Bangs, of New York, read a paper with the above title. He reported three cases of tuberculosis of the bladder, which he stated were typical of many, and in which the hygienic treatment was followed by very beneficial results. Among the many troublesome cases of disease of the bladder one was called upon to treat, none was more difficult than those due to tuberculous infection. At first the symptoms in these cases were often obscure and so similar to those produced by other morbid conditions that the diagnosis was frequently doubtful, and in many even impossible. Later on the clinical picture became so characteristic that all doubt as to the diagnosis was removed. But at this stage treatment could effect but little. But if the disease could be discovered in its incipiency and proper measures taken for its relief, it might be regarded as curable, certainly in the sense of being held in abeyance. Vitalization of tissue was what these patients needed, and they required at least two years of good hygienic residence in a temperate climate; and besides climate they needed occupation. Surgical traumatism produced by over zealous efforts to relieve local symptoms seemed to result in more harm than good. The author also reported three cases of malignant disease of the bladder, in which he stated that a cure also depended upon an early diagnosis. Unfortunately, in these cases, many of the early symptoms were overlooked or misunderstood. In conclusion, he stated that he had contrasted these two groups in order to present for discussion the points: (1) that cases of incipient tuberculosis of the bladder should be subjected to hygienic rather than to surgical treatment, and (2) that in the incipient stages of neoplasms surgical treatment of the most radical kind should be instituted. J. P. Bryson (St. Louis) said he was entirely in accord with the statement made by Bangs regarding the treatment of the tuberculous cases. In tuberculous cystitis perineal drainage was not to be recommended. In two of his cases the perineal wound became infected and failed to heal. The suprapubic route was altogether to be preferred when drainage is to be resorted to. In cases of vesical neoplasms the symptoms frequently came on late. If the new growth was situated toward the fundus, or at any considerable distance away from the vesical outlet, there was no reason why we should get any symptoms of its existence for a considerable length of time, and the first symptom was likely to be hemorrhage. G. W. Allen (Boston) reported a case of tuberculosis of the bladder in which the symptoms entirely disappeared under hygienic treatment.—*Tri-State Medical Journal.*

